# Genome‐wide association mapping reveals novel genes and genomic regions controlling root‐lesion nematode resistance in chickpea mini core collection

**DOI:** 10.1002/tpg2.20508

**Published:** 2024-09-13

**Authors:** Ashish Kumar, Yogesh Dashrath Naik, Vedant Gautam, Sunanda Sahu, Vinod Valluri, Sonal Channale, Jayant Bhatt, Stuti Sharma, R. S. Ramakrishnan, Radheshyam Sharma, Himabindu Kudapa, Rebecca S. Zwart, Somashekhar M. Punnuri, Rajeev K. Varshney, Mahendar Thudi

**Affiliations:** ^1^ Jawaharlal Nehru Krishi Vishwa Vidyalaya (JNKVV) Jabalpur Madhya Pradesh India; ^2^ Department of Agricultural Biotechnology and Molecular Biology Dr. Rajendra Prasad Central Agricultural University (RPCAU) Pusa Bihar India; ^3^ International Crops Research Institute for the Semi‐Arid Tropics (ICRISAT) Hyderabad Telangana India; ^4^ National Institute of Plant Health Management (NIPHM) Hyderabad Telangana India; ^5^ Centre for Crop Health and School of Agriculture and Environmental Science University of Southern Queensland (UniSQ) Toowoomba Queensland Australia; ^6^ College of Agriculture, Family Sciences and Technology, Agriculture Research Station Fort Valley State University Fort Valley Georgia USA; ^7^ WA State Agricultural Biotechnology Centre, Centre for Crop and Food Innovation Murdoch University Murdoch Western Australia Australia

## Abstract

Root‐lesion nematodes (RLN) pose a significant threat to chickpea (*Cicer arietinum* L.) by damaging the root system and causing up to 25% economic losses due to reduced yield. Worldwide commercially grown chickpea varieties lack significant genetic resistance to RLN, necessitating the identification of genetic variants contributing to natural resistance. This study identifies genomic loci responsible for resistance to the RLN, *Pratylenchus thornei* Sher & Allen, in chickpea by utilizing high‐quality single nucleotide polymorphisms from whole‐genome sequencing data of 202 chickpea accessions. Phenotypic evaluations of the genetically diverse set of chickpea accessions in India and Australia revealed a wide range of responses from resistant to susceptible. Genome‐wide association studies (GWAS) employing Fixed and Random Model Circulating Probability Unification (FarmCPU) and Bayesian‐Information and Linkage‐Disequilibrium Iteratively Nested Keyway (BLINK) models identified 44 marker‐trait associations distributed across all chromosomes except Ca1. Crucially, genomic regions on Ca2 and Ca5 consistently display significant associations across locations. Of 25 candidate genes identified, five genes were putatively involved in RLN resistance response (glucose‐6‐phosphate dehydrogenase, heat shock proteins, MYB‐like DNA‐binding protein, zinc finger FYVE protein and pathogenesis‐related thaumatin‐like protein). One notably identified gene (*Ca_10016*) presents four haplotypes, where haplotypes 1–3 confer moderate susceptibility, and haplotype 4 contributes to high susceptibility to RLN. This information provides potential targets for marker development to enhance breeding for RLN resistance in chickpea. Additionally, five potential resistant genotypes (ICC3512, ICC8855, ICC5337, ICC8950, and ICC6537) to *P. thornei* were identified based on their performance at a specific location. The study's significance lies in its comprehensive approach, integrating multiple‐location phenotypic evaluations, advanced GWAS models, and functional genomics to unravel the genetic basis of *P. thornei* resistance. The identified genomic regions, candidate genes, and haplotypes offer valuable insights for breeding strategies, paving the way for developing chickpea varieties resilient to *P. thornei* attack.

## Core Ideas

- Five accessions (ICC3512, ICC8855, ICC5337, ICC8950, and ICC6537) emerged as resistant to root‐lesion nematodes.
- Forty‐four marker‐trait associations for RLN resistance were identified.
- Genes like glucose‐6‐phosphate dehydrogenase (*G6PDH)*, heat shock proteins (*HSP*s), MYB‐like DNA‐binding protein, zinc finger FYVE protein, and PR‐related thaumatin‐like proteins are associated with RLN resistance.
- Haplotype analysis of candidate genes, particularly *Ca_10016*, revealed associations with RLN susceptibility, providing insights for further breeding efforts.

**Abbreviations**
ANOVA: analysis of variance
BLINK: bayesian‐information and linkage‐disequilibrium iteratively nested keyway
BLUE: best linear unbiased estimate
E1: Experiment 1
E1‐2: Experiment 1 and 2 combined
E1–3: Experiment 1–3 combined
E2: Experiment 2
E3: Experiment 3
FarmCPU: fixed and random model circulating probability unification
*G6PDH*
: glucose‐6‐phosphate dehydrogenase
GWAS: genome‐wide association studies
JNKVV: Jawaharlal Nehru Krishi Vishwavidyalaya
MTAs: marker‐trait associations
PC: principal component
PCA: principal component analysis
QTL: quantitative trait loci
RF: reproduction factor
RLN: root‐lesion nematodes
SNP: single nucleotide polymorphism

## 1 INTRODUCTION

Chickpea (*Cicer arietinum* L.) is cultivated in more than 55 countries across the globe and stands as the third most extensively grown food legume in terms of harvested area, after soybean and dry beans (Food and Agriculture Organization of the United Nations [FAOSTAT], 2022). Chickpea is generally found in two main types: kabuli and desi. Typically, kabuli chickpeas have a beige outer coat and are large, smooth seeds without edges. In contrast, desi chickpeas have a dark‐colored coat and are small, rough seeds. Both types of chickpeas face a range of similar challenges, including biotic stresses arising from bacteria, fungi, nematodes, viruses, and insects. The root‐lesion nematodes (RLN), *Pratylenchus thornei* Sher & Allen, are emerging as a serious threat to chickpea production in India as well as the other part of the world due to its wide host range and adaptability to various climatic conditions. It has been identified as the cause of significant yield losses globally in chickpea of 25% (Kumar et al., 2022; Thompson & Clewett, 2021; Zwart et al., 2019), as well as yield reductions ranging from 10% to 28% in various other crop plants (Kumar et al., 2021; Zwart et al., 2019). Di Vito et al. (1992) found that as few as 31 *P. thornei* per liter of soil can harm chickpea in Syria, with 2000 *P. thornei* per liter causing up to 58% yield loss.

Infestation by *Pratylenchus* spp. is characterized by the formation of lesions, necrotic regions, browning of tissues, and subsequent cellular death (Pulavarty et al., 2021). RLN feed and move within the root cortical tissue, leading to cell damage and decreased root growth. This impairment in root function leads to reduced water and nutrient uptake, which ultimately impacts crop yield (Zwart et al., 2019). These nematodes are migratory endoparasites and can move between the plant roots and soil. The population density of RLN builds up in the soil when susceptible crops are grown, causing damage not only to the current crop but also subsequent crops (Owen et al., 2014). Nematodes also play a pivotal role in the emergence of diseases triggered by soil‐borne pathogens in chickpea and various crops (Back et al., 2002). They act as vectors or facilitators, contributing to the spread of these diseases that ultimately negatively impact agricultural production (Bhatt & Vadhera, 1997; Castillo et al., 1998). The use of chemical nematicides has been effective in controlling nematode populations in soil. Moreover, nematicides have detrimental effects on the environment and the potential to harm non‐target organisms and ecosystems (Sasanelli et al., 2021). Therefore, control of RLN is reliant on the use of resistant crops in rotation sequences (Thompson et al., 2020) with two or more consecutive resistant crops required to reduce population densities to be below damaging level (Owen et al., 2014). The broad host range of RLN across many cereal and pulse crops, including chickpea, necessitates the development of new crop varieties with high levels of genetic resistance to increase yield and minimize economic losses.

During the last decade, a plethora of high‐throughput, low‐cost genotyping and sequencing technologies have enabled the sequencing of several genomes, including draft genomes, as well as enhancing our understanding of the genetics of various biotic and abiotic stresses (Thudi et al., 2023; Varshney et al., 2021). The genome‐wide association studies (GWAS) approach has been extensively deployed in recent years for identifying the genomic loci responsible for various biotic stresses in different crop plants (Gangurde et al., 2022). The chickpea reference set, comprising 300 genotypes representing global chickpea diversity, has been used for dissecting complex traits through GWAS, including drought, heat, and phosphorus use efficiency (Jha et al., 2021; Thudi et al., 2021, 2014; Varshney et al., 2019).

In the context of climate change, to mitigate the yield losses due to RLN, identification of genomic loci or the genes responsible for resistance to *P. thornei* is fundamental for chickpea crop improvement. The lack of effective resistance in existing commercial chickpea varieties has prompted research efforts to search for novel sources of resistance in diverse international germplasm collections of landrace chickpea against *P. thornei* (Channale et al., 2023) and wild *Cicer* species, which were the progenitors to *C. arietinum*, against *P. thornei* (Reen et al., 2019) and *P. neglectus* (Rostad et al., 2022). Two studies from Australia, one based on GWAS and the other based on quantitative trait loci (QTL) mapping, have reported genomic regions contributing to *P. thornei* resistance in *C. arietinum* (Channale et al., 2023; Khoo et al., 2021). However, to date, no studies have reported the genomic regions responsible for RLN resistance from India, where significant yield losses are reported due to RLN in chickpea. In the present study, we report significant marker‐trait associations (MTAs) and genes responsible for RLN resistance deploying high‐quality single nucleotide polymorphisms (SNPs) reported earlier (Varshney et al., 2019), and the phenotyping data generated on the chickpea reference set in India, as well as data generated by Channale et al. (2023) in Australia. MTAs and identified genes are not only useful for breeding purposes but also provide the molecular basis of nematode resistance.

**Core Ideas**

- Five accessions (ICC3512, ICC8855, ICC5337, ICC8950, and ICC6537) emerged as resistant to root‐lesion nematodes.
- Forty‐four marker‐trait associations for RLN resistance were identified.
- Genes like glucose‐6‐phosphate dehydrogenase (*G6PDH)*, heat shock proteins (*HSP*s), MYB‐like DNA‐binding protein, zinc finger FYVE protein, and PR‐related thaumatin‐like proteins are associated with RLN resistance.
- Haplotype analysis of candidate genes, particularly *Ca_10016*, revealed associations with RLN susceptibility, providing insights for further breeding efforts.

## 2 EXPERIMENTAL PROCEDURES

### 2.1 Plant material

The association panel consisted of 202 chickpea accessions (Table S1) obtained from the chickpea reference set (Upadhyaya et al., 2008). Among these genotypes, 154 were of the desi type, 40 were of the kabuli type, and eight were pea‐shaped (including seven breeding lines, 193 landraces, and two advanced cultivars). The association panel contain accessions from different geographical origins. However, a large number of accessions originate from India (40.6%) and Iran (25.8%).

### 2.2 Phenotypic evaluation of chickpea genotypes to *P. thornei* resistance

Chickpea genotypes were grown in a completely randomized design with five replicates. A total of two controlled greenhouse experiments were conducted in India to assess the response of the association panel to *P. thornei* inoculation. Experiments 1 and 2 (E1 and E2) were grown at Jawaharlal Nehru Krishi Vishwavidyalaya (JNKVV), Jabalpur, Madhya Pradesh (23°12′51″ N 79°57′41″ E) during 2020–2021 and 2021–2022, respectively. The accessions were phenotyped under controlled greenhouse conditions in earthen pots filled with a mixture of 500 g of sterilized soil and sand in a 3:1 ratio. Initially, two seeds per pot were sown to ensure one plant per pot. At the time of sowing, three sterilized glass rods with a diameter of 5 mm each were inserted around the seed, reaching a depth of 4 cm into the soil.

One thousand nematodes were inoculated onto each 7‐day‐old chickpea seedling by exposing the root surface after removing the glass rod from pots. After 35 days, roots were extracted from the soil, and nematodes were individually counted in both the roots and the soil using Cobb's sieving and decanting method (Cobb, 1918). The collected nematode suspensions were stored at 4°C, and the number of nematodes in 1 mL samples was counted under a compound microscope at 40x magnification. RLN response can be predicted as the reproduction factor (RF; RF = Pf/Pi, where Pf = final nematode population and Pi = initial nematode population) as an indicator of susceptibility (Pf/Pi > 1) or resistance (Pf/Pi < 1) (Seinhorst, 1967) or alternatively, as number of nematodes per unit of root and/or soil (Fatemi & Jung, 2023; Reen et al., 2019).

A third experiment (E3) was included in this study by utilizing phenotypic data published by Channale et al. (2023). Channale et al. (2023) phenotyped a larger set of chickpea genotypes, which included all 202 genotypes investigated in this study, for resistance to *P. thornei* in two experiments grown in controlled environment glasshouses at the University of Southern Queensland, Australia (−27°36′15″ S 151°55′55″ E). For each experiment, pots were arranged in a randomized row‐column design with three replicates. The phenotyping methodology differed from that described for E1 and E2 with plants grown in pots containing 330 g (oven‐dried equivalent) of pasteurized black Vertosol soil (Isbell, 1996) and inoculated with 3300 nematodes/pot. Plants were grown for 18 weeks. The soil and roots of each pot were mixed thoroughly, and nematodes were extracted from a 150 g subsample using the Whitehead tray method (Whitehead & Hemming, 1965). Enumeration of nematodes was performed using a Peters counting chamber of 1 mL capacity (Chalex Corporation) under a compound microscope (Olympus BX53). *Pratylenchus thornei* counts were transformed by log_e_ (*x* + 1), where *x* = number of *P. thornei*/kg of soil and roots. Nematode count data were transformed to fulfill the assumptions of homogeneity of variance and normal distribution, prior to statistical analysis. The duplicated experiments were analyzed together using a linear mixed model to obtain best linear unbiased estimates (BLUEs).

### 2.3 Statistical analysis

Analysis of variance (ANOVA) was carried out using the R package “variability” (Popat et al., 2020). Meta‐R software was used to estimate BLUEs values for the random effects (Alvarado et al., 2020). We used Microsoft Excel for Bland–Altman (B–A) analysis to check the agreement between datasets. In this analysis, the “bias” or mean difference between the two datasets was calculated. Additionally, the limit of agreement, defined as the mean difference ± 1.96 times the standard deviation of the differences (Bland & Altman, 1986), shows the range within which most differences lie. If data points are close to the zero line in the plot, it indicates good agreement between the methods.

### 2.4 Genome‐wide association analysis

GWAS was performed by analyzing each phenotyping dataset alone and then combined. In total, there were five distinct phenotyping datasets used for GWAS analysis: (1) E1; (2) E2; (3) Experiment 1 and 2 combined (E1‐2); (4) E3; (5) Experiments 1–3 combined (E1–3). The SNP genotyping dataset used in the study was a subset of the chickpea accessions genotyped by Thudi, Chitikineni, et al. (2016a), Thudi, Khan, et al. (2016b) and Varshney et al. (2019). GWAS analysis was conducted using two statistical models, Bayesian‐Information and Linkage‐Disequilibrium Iteratively Nested Keyway (BLINK) and Fixed and Random Model Circulating Probability Unification (FarmCPU), in the GAPIT3 package of R (Lipka et al., 2012). The BLINK and FarmCPU models effectively control false positives in GWAS analysis. FarmCPU conducts marker tests using associated markers as covariates in a fixed‐effect model and independently optimizes these covariates in a random effect model. In contrast, BLINK improves efficiency by eliminating FarmCPU's assumption that causal genes are evenly distributed across the genome, thus removing the need to optimize marker size and number. Considering these advantages to control false positives, we performed GWAS analysis using these models on each dataset generated independently as well as on combined datasets. Principal component analysis (PCA) was used to validate population stratification with GAPIT3. Our analysis included a total of 476,299 SNPs that met the following criteria: minor allele frequency ≤0.05, missing data rate ≤0.20, and minimum heterozygosity ≤0.10, as filtered out by VCFtools. To address the issue of false positives, we employed the Bonferroni test as a correction method in the GWAS analysis. Using the Bonferroni correction at a significance level of 5% [−log (0.05/number of variants)], we established the threshold value as −log10(*p*) = 6.97. Additionally, the suggestive threshold was calculated to increase the true positive discovery of MTAs. The suggestive threshold, as defined by Lander and Kruglyak (1995), represents the expectation of one false positive per genome scan under the null hypothesis.

### 2.5 Candidate gene and haplotype identification

Candidate genes were identified by extracting SNP annotations from the SnpEff output file and obtaining corresponding functional gene annotations from the Phytozome database (https://phytozome‐next.jgi.doe.gov/). SNPs present in the genic region of annotated genes were extracted for all the genotypes included in the study. The genotypes were further analyzed for presence of missing or heterozygous calls for the SNPs in the genic region. For a given gene, genotypes with missing SNPs or possessing heterozygous calls were excluded. Later, haplotypes were identified based on the SNP alleles for remaining genotypes by concatenating to form a sequence. The haplotype obtained from each genotype and candidate gene was sorted to find unique haplotypes. These unique haplotypes were named as Hap1, Hap2, …, HapN. Further, the haplotype count was calculated based on the number of genotypes with each haplotype.

## 3 RESULTS

### 3.1 Genotypic variability and phenotypic evaluation of *P. thornei* resistance

**FIGURE 1:**
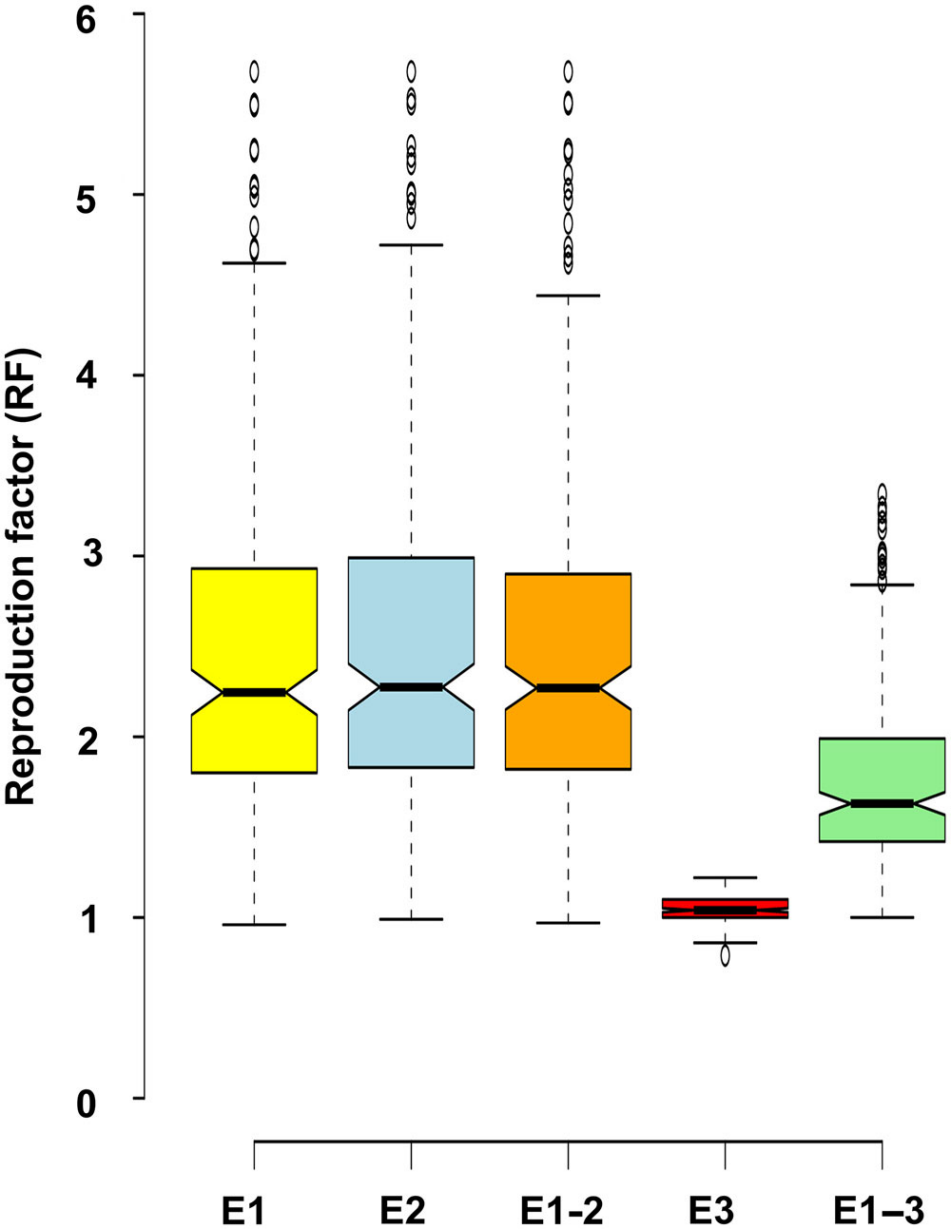
Distribution pattern of calculated reproduction factors of nematodes in chickpea germplasm. This figure illustrates the distribution pattern of calculated reproduction factors of nematodes in chickpea germplasm phenotyped at Jabalpur during 2020–2021 (Experiment 1 [E1]) and 2021–2022 (Experiment 2 [E2]), pooled seasons (Experiment 1 and 2 combined [E1‐2]), Australia (Experiment 3 [E3]), and combined Jabalpur and Australia (Experiment 1–3 combined [E1–3]) datasets.

**FIGURE 2:**
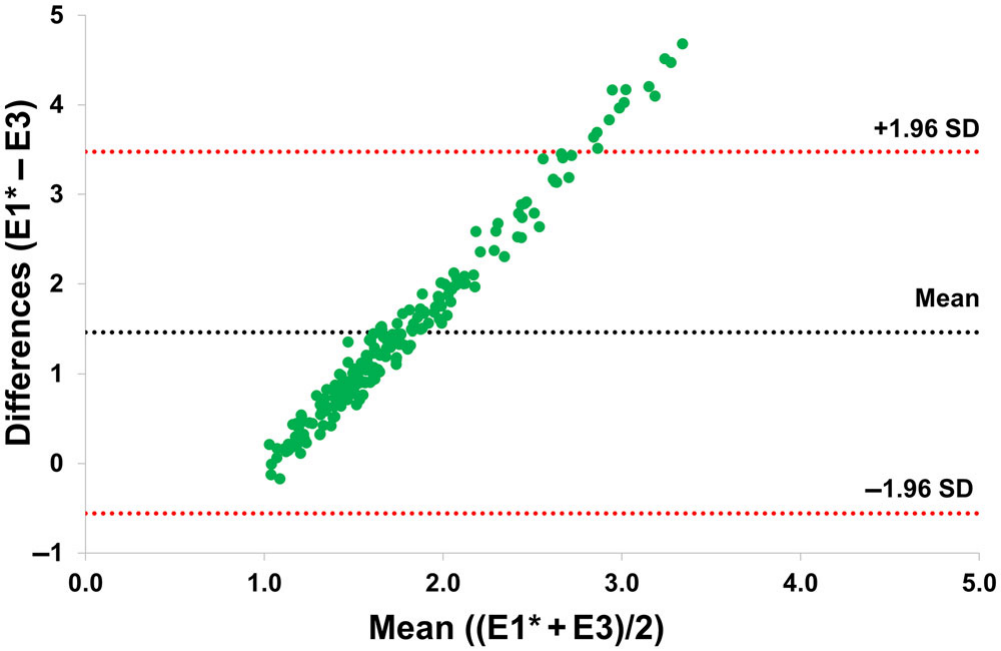
Bland–Altman correction between different datasets. The plot shows a scatter plot with the differences between two datasets on the *y*‐axis and their averages on the *x*‐axis. A black line indicates the mean difference, while red lines indicate ± 1.96 standard deviations to highlight potential outliers. It represents the correlation between the Experiment 1–2 (E1*) and Experiment 3 (E3) datasets. The mean difference between all combinations of the two datasets is not near zero, indicating a poor agreement between nematode responses in different locations. This suggests that there are notable variations in the measurements taken at these locations, which may indicate differences in the number of nematodes in the inoculum while conducting different experiments.

ANOVA indicated a significant genetic variance (*p* < 0.001) in the association panel, with a substantial range of variation in *P. thornei* reproduction across all experiments (Table S2). The broad‐sense heritability was observed to be above 0.97 in both E1 and E2, and the coefficient of variation was recorded as 2.5 and 2.4 for E1 and E2, respectively (Table S2). The RF values ranged from 0.96 to 5.68 for E1 and from 0.99 to 5.68 for E2. Further, the mean pooled values of E1 and E2 (E1‐2) ranged from 0.97 to 5.68, with an average of 2.50. The RF based on combined datasets from all experiments (E1–3) revealed a substantial variation, ranging from 1.00 to 3.34, with an average of 1.77 (Figure 1; Table S3). In Jabalpur experiments (E1, E2, and E1‐2), two germplasm lines (ICC3512 and ICC8855) exhibited resistance to *P. thornei*. The RF based on Australian datasets (E3) ranged from 0.79 to 1.22, with an average of 1.04. When the data from both locations, India and Australia (E1–3), were combined, a single‐resistant genotype (ICC2242) was identified (Table S3), while genotypes such as ICC12928, ICC12654, ICC12824, and ICC12851 showed high susceptibility to RLN. To compare the agreement between the different datasets, we performed the B–A correlation analysis. B–A correlation analysis revealed poor agreement between the two datasets (E1‐2 and E3) for *P. thornei* reproduction (Figure 2). Zero or near‐zero bias (bias refers to the mean difference between two sets of measurements) shows no or less systematic difference between datasets, while positive or negative bias indicates one method gives higher or lower values on average. The analysis showed a bias of 1.45, with limits of agreement from 3.47 to −0.55 (Figure 2). These results indicate that the datasets compared for nematode responses show significant differences.

### 3.2 SNPs and population stratification

A comprehensive set of 476,299 high‐quality SNPs based on sequence data generated earlier provided an extensive genome coverage, averaging about 59,537 SNPs per chromosome, or approximately one variant every 728 bp. Chromosome 4 (Ca4) exhibited the highest SNP count, representing 34.4% of the total, while the least number of SNPs were found on Ca8 (18,890 variants) (Table S4). As the number of subpopulations using Admixture model in STRUCTURE was inconclusive, PCA was used to determine the population structure. The first four principal components (PCs) collectively explained 62% of the total genetic variance. PC1 accounted for approximately 23.37%, PC2 for 14.68%, PC3 for 11.61%, and PC4 for 8.74%. The first 10 PCs indicated an inflection point at PC2, suggesting that the population structure primarily hinged on the first two PCs (Table S5). Therefore, FarmCPU and BLINK employed the two PCs as covariates for GWAS analysis to correct for population stratification.

### 3.3 Genome‐wide association mapping for *P. thornei* resistance

**FIGURE 3:**
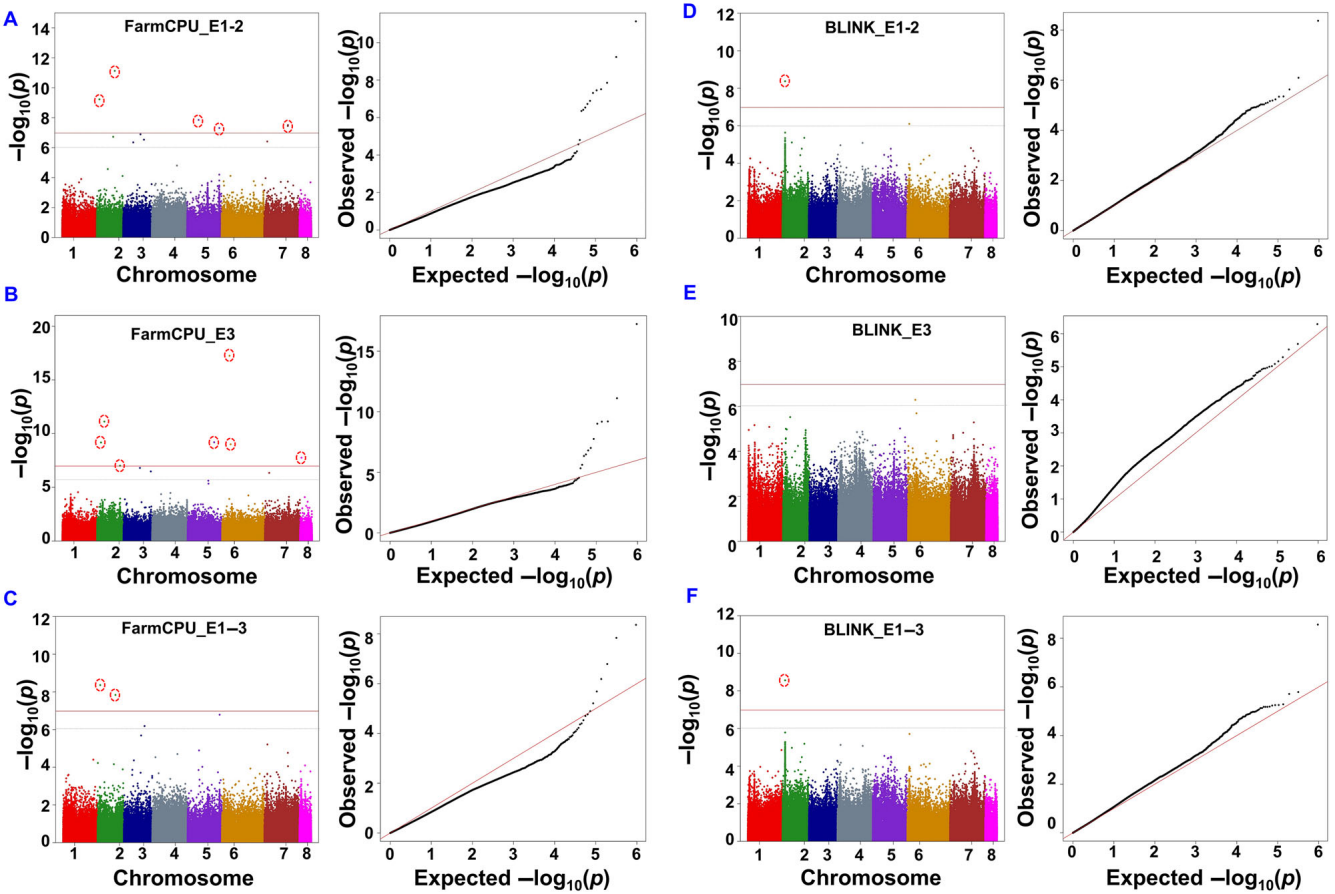
Manhattan and quantile–quantile (*Q*−*Q*) plots of genome‐wide association studies (GWAS) signals for nematode response using Fixed and Random Model Circulating Probability Unification (FarmCPU) and Bayesian‐Information and Linkage‐Disequilibrium Iteratively Nested Keyway (BLINK) Model. This figure presents Manhattan plots and QQ plots (A, B, and C) illustrating GWAS results for nematode response in Jabalpur (E1‐2), Australia (E3), and combined Jabalpur and Australia (E1–3) using the FarmCPU model. Also presents Manhattan plots and *Q*−*Q* plots (D, E, and F) illustrating GWAS results for nematode resistance in E1‐2, E3, and E1–3 datasets using the BLINK model. In each plot, the red line signifies the GWAS significance (Bonferroni threshold), with significant marker‐trait associations (MTAs) found above this threshold shown in red circles, while the gray line represents the suggestive significance threshold. On the right side of each Manhattan plot, the corresponding *Q*−*Q* plot is provided, comparing observed and expected *p*‐values from GWAS results.

The distribution of MTAs across E1, E2, E1‐2, E3, and E1–3 datasets varied. In total, we identified 44 MTAs on all chromosomes except Ca1, with −log10 (*p*) ≥ 6 (Table S6). Most MTAs (13) were identified in the E1‐2. Further, we detected the least MTAs (5) when E1–3 dataset was analyzed. The observed *p*‐values ranged from 5.70 × 10^−18^ to 8.88 × 10^−07^, underlining the statistical significance of SNP associations with *P. thornei* resistance. Among the 44 MTAs, 28 had *p*‐values above the Bonferroni correction threshold [−log10 (*p*) ≥ 6.9]. Most of these were observed in E1‐2 and E3 (7), while fewer were observed for E1–3 (3) using both models (Figure 3). Overall, FarmCPU detected 37 MTAs across all eight chromosomes except Ca1, while BLINK identified seven MTAs on Ca2 and Ca3 (Table S6). Among seven MTAs identified using BLINK, 57.14% were present on Ca2 and 42.85% on Ca6. Nevertheless, like the BLINK model, FarmCPU also provided the majority (32.43%) of MTAs on Ca2, followed by Ca3 (21.62%), Ca5 (18.91%), and Ca7 (16.21%), while single MTA was on Ca8 (2.70%). Five of these MTAs were common to both models, and two were unique to BLINK (Table S6). When data generated from E1 and E2 were analyzed independently, we identified five and six significant [−log10 (*p*) ≥ 6.9] MTAs, respectively. Among 28 significant MTAs identified based on phenotypic data generated in all experiments, the SNP loci (Ca2_3571754 and Ca6_10213780) were identified in both BLINK and FarmCPU models (Table 1). Interestingly, the SNP locus (Ca2_3571754) on Ca2 was found associated with calculated RFs of nematodes based on data generated in all seasons in India independently as well as all data combinations except when E3 data are analyzed alone (Table 1). The quantile–quantile (*Q*−*Q*) plot demonstrated a noticeable deviation between observed and expected *p*‐values, especially for markers strongly associated, indicating effective control of confounding effects during analysis (Figure 3).

**TABLE 1:** Summary of significant single nucleotide polymorphism (SNP) loci and putative genes identified across various models and experiments.

	Model		Environment/location					Gene ID	Gene function
SNP loci	FarmCPU	BLINK	E1	E2	E1‐2	E3	E1–3		
Ca2_10110663	Y	N	N	N	N	Y	N	*Ca_20377*	NOC2P family protein
Ca2_24904377	Y	N	Y	Y	Y	N	Y	*Ca_24904*	GAG‐POL‐related retrotransposon
Ca2_31860491	Y	N	N	N	N	Y	N	*Ca_17832*	Zinc finger FYVE domain containing protein
Ca2_3571754	Y	Y	Y	Y	Y	N	Y	*Ca_10505*	KH domain containing RNA binding protein
Ca2_5358519	Y	N	N	N	N	Y	N	*Ca_14672*	Glucose‐6‐Phosphate 1‐Dehydrogenase
Ca3_24194574	Y	N	N	Y	Y	N	N	*Ca_06100*	Lysine tRNA ligase like
Ca3_29050880	Y	N	Y	N	Y	N	Y	*Ca_07210*	–
Ca5_16094293	Y	N	Y	N	Y	N	N	*Ca_17909*	Telomerase reverse transcriptase
Ca5_37053082	Y	N	N	N	N	Y	N	Ca_01510	Small seven transmembrane domain‐containing protein
Ca5_44908084	Y	N	N	Y	Y	N	Y	*Ca_03935*	EAMA‐Like transporter family
Ca6_10213780	Y	Y	N	N	N	Y	N	*Ca_08568*	Translation initiation factor EIF‐2B subunit Gamma
Ca6_11810044	Y	N	N	N	N	Y	N	*Ca_24838*	Retrotransposon GAG Protein
Ca6_2965425	Y	Y	Y	N	Y	N	N	*Ca_10438*	Protein tyrosine kinase
Ca7_32774901	Y	N	Y	N	Y	N	N	*Ca_10016*	MPPE1‐like protein
Ca7_32943172	Y	N	N	Y	N	N	N	*Ca_10004*	Nodulating signaling pathway 1 protein
Ca7_32966636	Y	N	N	N	Y	N	N	*Ca_10001*	Guanosine‐3′,5′‐BIS Diphosphate 3′‐Pyrophosphohydrolase
Ca8_1865048	Y	N	N	N	N	Y	N	*Ca_02417*	MYB‐like DNA‐binding protein

*Note*: “Y” indicates that the SNP locus was found to be associated with the reproduction factor based on a dataset generated at a particular location or using a given GWAS model, while “N” indicates that the SNP locus was not found to be associated with the trait at a particular location. “E1” denotes the dataset generated from Experiment 1, conducted at JNKVV, Jabalpur, during 2020–2021; “E2” denotes the dataset generated from Experiment 2, conducted at JNKVV, Jabalpur, during 2021–2022; “E3” denotes the dataset generated by Channale et al. (2023) at Toowoomba, Australia; “E1‐2” denotes the mean performance of chickpea genotypes from the combined datasets of Jabalpur experiments; and “E1–3” denotes the mean performance of chickpea genotypes from the combined datasets of Jabalpur and Australia.

Abbreviations: BLINK, Bayesian‐Information And Linkage‐Disequilibrium Iteratively Nested Keyway; FarmCPU, Fixed and Random Model Circulating Probability Unification; SNP, single nucleotide polymorphism.

### 3.4 Candidate genes and haplotypes for RLN resistance

**FIGURE 4:**
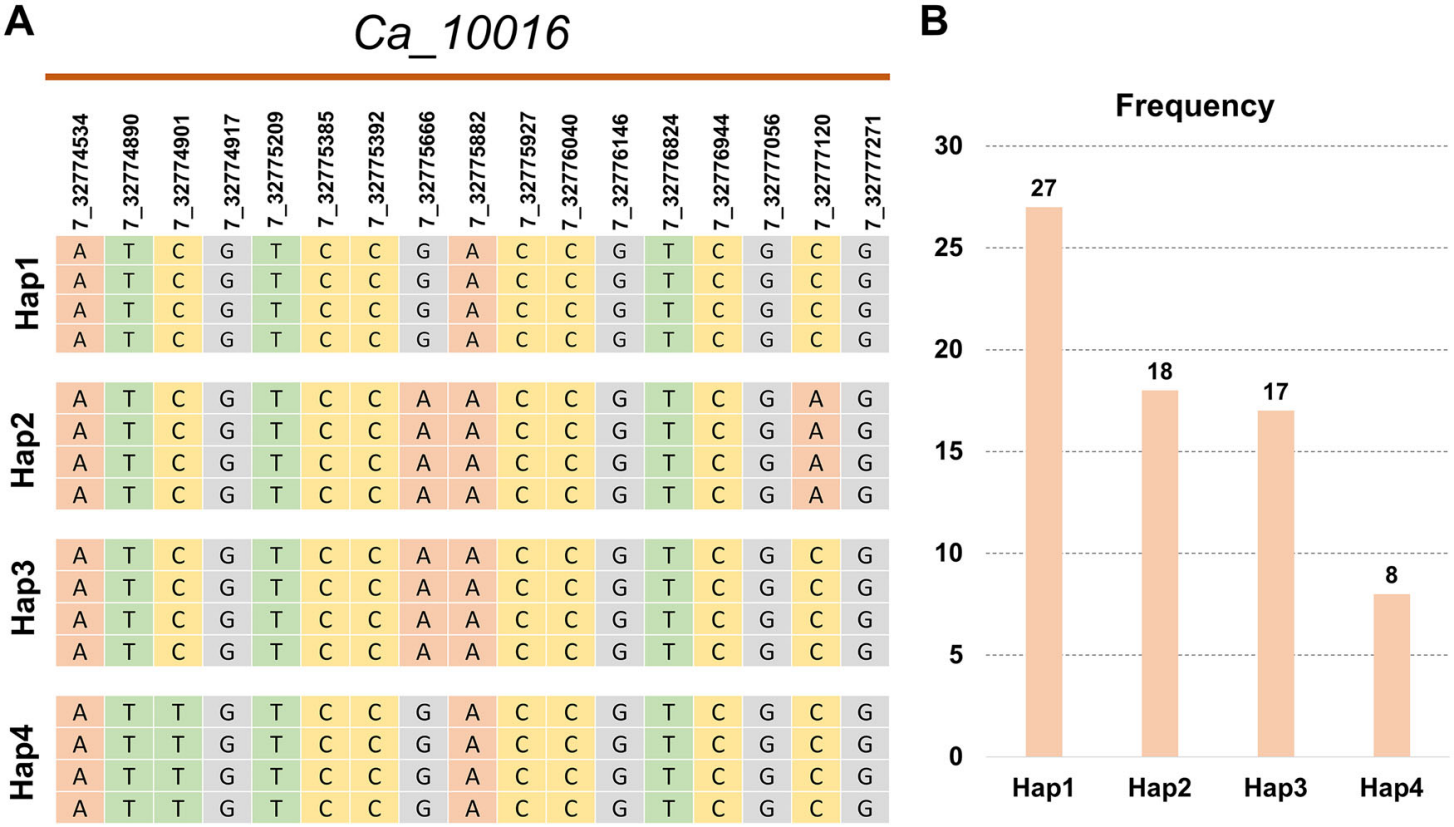
Haplotypes (Hap) for the gene *Ca_10016* encoding metallophosphoesterase present on Ca7. A total of four haplotypes were identified based on 70 genotypes. Part A represents the sequence of the four haplotypes, and Part B shows the number of genotypes in each haplotype. Hap4, which includes eight genotypes, may be responsible for high susceptibility to root‐lesion nematodes (RLN).

Based on the physical position of the MTA/SNP locus, we found that most of the MTAs (80%) were in intergenic regions, while five were located in genes, either in intronic or exonic regions. Upon functional annotation using the Phytozome database, we obtained functions for 24 out of 25 genes (Table S7). Ca2 harbored the majority of the annotated genes. Further, on Ca2, the following genes were present: *Ca_20377* (encodes NOC2P family protein), *Ca_14990* (encodes RHO GTPASE‐activating protein REN1 ISOFORM X5), *Ca_24904* (encodes GAG‐POL‐related retrotransposon), *Ca_17832* (encodes zinc finger FYVE domain containing protein), *Ca_10505* (encodes KH domain containing RNA binding protein), and *Ca_14672* (glucose‐6‐phosphate 1‐dehydrogenase). Additionally, five genes were identified on Ca7: *Ca_10001* (encodes guanosine‐3′,5′‐bis diphosphate 3′‐pyrophosphohydrolase), *Ca_10016* (encodes MPPE1‐like protein), *Ca_03058* (encodes heat shock protein), *Ca_06764* (encodes ATPase 6, plasma membrane‐type), and *Ca_10004* (nodulation‐signaling pathway 1 protein) (Table S7). In an earlier study based on multi‐locus association mapping, genes including receptor‐linked kinases (Ca1, Ca4, and Ca6), GDSL‐like lipase/acylhydrolase (Ca3), aspartic proteinase‐like and thaumatin‐like protein (on Ca4), AT‐hook DNA‐binding, and HSPRO2 (on Ca6) were reported as candidate genes for *P. thornei* resistance in the chickpea reference set (Channale et al., 2023). Haplotype analysis was performed for 11 genes. Owing to missing SNP calls or heterozygous calls in the genes, we identified four haplotypes in only one gene*, Ca_10016*, on Ca7. For this gene, a total of 70 genotypes had the complete gene sequence information without missing SNP calls or heterozygous calls at a given locus. Among 70 genotypes, four genotypes were resistant, 61 genotypes were susceptible and five were highly susceptible, which contain the Hap4 (Figure 4; Table S8).

## 4 DISCUSSION

In the three experiments performed under controlled conditions at JNKVV, Jabalpur in India, and Toowoomba, Australia, wide variation in genotypic response to RLN inoculation was observed among the genotypes of the chickpea association panel. This variation from resistant to susceptible underscores diverse resistance and susceptibility levels within the collection. In general, the correlation between data generated between two different methods/instruments/locations is always misleading and should not be used for assessing their comparability. The B–A comparison is the simplest method to evaluate a bias between the mean differences and estimate an agreement interval, within which 95% of the differences of the second method fall, compared to the first one. The B–A analysis is a method used to assess agreement between two quantitative measurements. We found poor agreement between nematode responses in different locations, which suggests that there are notable variations in the measurements taken at these locations, which may indicate differences in number of nematodes in the inoculum while conducting different experiments.

In the present study, we identified resistant genotypes, such as ICC3512, ICC8855, ICC5337, ICC8950, and ICC6537. Among these, two genotypes (ICC3512 and ICC8855) showed resistance in phenotyping experiments conducted in India, while three genotypes (ICC5337, ICC8950, and ICC6537) showed resistance in the phenotyping experiments conducted in Australia. These genotypes are promising candidates for further investigations aimed at identifying specific resistance‐conferring genes or genomic regions. This resistance may be due to the presence of stress‐responsive genes or the high expression of identified genes in response to nematodes. Based on the earlier screening of chickpea reference set at Toowoomba, Australia ICC8950, ICC5135, ICC6816, ICC95, and ICC14831 were reported as resistant to *P. thornei* (Channale et al., 2023). The identification of different sources of resistance could be attributed to the strain differences of *P. thornei*. Furthermore, on screening 600 germplasm lines at ICRISAT (International Crops Research Institute for the Semi‐Arid Tropics), Hyderabad, and IIPR (Indian Institute of Pulses Research), Kanpur, India, no resistant line was identified (Ali & Ahamad, 2000). Nevertheless, Thompson and colleagues (2011) reported one resistant genotype on screening a total of 453 germplasm lines from ICARDA (International Center for Agricultural Research in the Dry Areas), ICRISAT, and Australia.

With advancements in high‐throughput sequencing, GWAS has become a powerful tool for dissecting complex traits in chickpea (Farahani et al., 2022; Ravelombola et al., 2020; Thudi et al., 2021, 2023). Our study employed two multi‐locus GWAS models, FarmCPU and BLINK, to overcome limitations associated with single‐locus models (Li et al., 2018). FarmCPU outperformed compared to the BLINK model in controlling *p*‐value inflation and identifying new associated markers. Population structure corrections, through kinship and PCs, effectively mitigated confounding effects. Of the 44 MTAs identified, 28 originated from Indian datasets, and 16 were either individual Australia or combined datasets. Earlier MTAs were reported on all chromosomes of chickpea except Ca8 (Channale et al., 2023). Similarly, in this study, we identified only single GWAS signals on Ca8 and confirmed that Ca8 may not be significantly involved in RLN resistance. The SNP loci detected in more than one experiment (Ca2_3571754) using both models should be prioritized as targets for validation and functional analysis studies. The differences between our results and those of Channale et al. (2023) are likely due to using different GWAS models, including more chickpea lines in their study, and the varying lengths of the experiments—35 days in India versus 18 weeks in Australia. These factors influenced the results.

Significant SNPs were annotated using the Phytozome database, revealing 24 putative candidate genes. Notably, several genes play a significant role in disease resistance. The identified genes, including those encoding glucose‐6‐phosphate dehydrogenase (*G6PDH*), heat shock proteins (HSPs), MYB‐like DNA‐binding protein, zinc finger FYVE protein, and pathogenesis‐related thaumatin‐like proteins, are known for their roles in plant‐nematode responses. *G6PDH*, a key enzyme in the oxidative pentose phosphate pathway, contributes to basal defense against nematodes through NADPH (nicotinamide adenine dinucleotide phosphate) production and reactive oxygen species signaling. In the case of *Arabidopsis thaliana*, the loss of cytosolic *G6PDH*s reported to increase susceptibility to root‐knot nematode (*Meloidogyne* spp.) infection (Hu et al., 2019). Similarly, MYB transcription factors play crucial roles in various plant processes, including stress responses, metabolism, and defense (Biswas et al., 2023). For instance, in *Arabidopsis*, the *AtMYB96* gene is vital for ABA (abscisic acid) signaling and disease resistance (Seo & Park, 2010). It is also reported that overexpression of *AtMYB59* has enhanced the resistance of Arabidopsis to nematodes (Wiśniewska et al., 2021). Additionally, a previous study found that *CsMYB* genes were highly expressed in cucumber inoculated with *Meloidogyne incognita*, highlighting the important role of these transcription factors in regulating the resistance response (Cheng et al., 2020). Plant zinc finger proteins constitute a large protein family primarily associated with abiotic stress tolerance. However, in wheat, zinc finger RING/FYVE/PHD protein have been reported to play a role in the nematode resistance response (Chaturvedi et al., 2023; Singh et al., 2023).

HSPs strengthen tolerance to stresses, including nematode infestations (Hishinuma‐Silva et al., 2020). Thaumatin‐like proteins are associated with systemic‐acquired resistance and plant disease resistance (Wang et al., 2022). The gene *Ca_10505*, present on Ca2, encoding a KH domain containing RNA‐binding proteins, was reported to coordinate with microRNAs to regulate root‐knot nematode (*Caenorhabditis elegans)* development (Haskell & Zinovyeva, 2021). This may be implicated in the presence of the *Ca_10505* gene, which may enhance the susceptibility to RLN. On supplementation of jasmonic acid, the genes of chalcone reductase and shikimate O‐hydroxycinnamoyltransferase (*Ca_18222* gene) involved in flavonoid biosynthesis were reported to be upregulated in plants (Rahman et al., 2020). This is an interesting finding and links to earlier work in wheat where chalcone was identified as a metabolite differentially expressed in resistant and susceptible wheat (Rahman et al., 2020). The compound 12‐oxo‐Phytodienoic acid is a primary precursor of jasmonic acid that plays a role in regulating plant defense against nematodes in *Arabidopsis* (Gleason et al., 2016). The gene *Ca_06100* encodes for Lysine‐tRNA ligase that ensures the accurate pairing of lysine with its corresponding tRNA molecule. This charged tRNA molecule is then involved in the process of translation, allowing for the synthesis of proteins with the correct amino acid sequence. This fundamental process is essential for the proper functioning of cells and the overall growth and development of plants (Alberts et al., 2002). The gene *Ca_17909*, which encodes telomerase reverse transcriptase responsible for maintaining the length of telomeric DNA and ensuring chromosomal stability, could contribute to the overall fitness and resilience of plant cells (Shakirov et al., 2022).

In a recent study, Khoo et al. (2021) reported QTLs on Ca4 and Ca7 using a RIL (recombinant inbred line) population derived from PBA Hatrick and Kyabra. Further, they also reported a total of 69 genes within the QTL (1.03 Mb region from 22.57 to 23.60 Mb) on the Ca7. In our study, among six genes we identified on Ca7, three genes were in the 0.19 Mb region (located between 32.77 and 32.96 Mb). The genomic region identified in the present study is 9.17 Mb away from the earlier report (Khoo et al., 2021). This is quite possible, as the earlier study used the RIL population derived from two contrasting parents for QTL analysis, while we used the diverse chickpea collection for GWAS analysis. In the present study, we identified a total of four haplotypes in a set of 70 genotypes that possess the complete allele calls for the gene *Ca_10016* on chromosome Ca7. The gene *MPPE1* (metallophosphoesterase 1) is a protein‐coding gene involved in hydrolase activity and phosphoric diester hydrolase activity. The haplotype 1 (27 genotypes), 2 (18 genotypes), and 3 (17 genotypes) are responsible for moderate susceptibility to *P. thornei* in chickpea. While the haplotype 4 (8 genotypes) could be responsible for high susceptibility to RLN. In an earlier report, on studying the transcriptomes of control and inoculated roots of three chickpea genotypes, namely, D05253  >  F3TMWR2AB001 (a resistant advanced breeding line), PBA HatTrick (a moderately resistant cultivar) and Kyabra (a susceptible cultivar), at 20 and 50 days post‐inoculation, the genes encoding hydrolase activity were upregulated in case of resistant genotype, D05253  >  F3TMWR2AB001 (Channale et al., 2021). *MPPE1* is a key gene for which markers should be designed and validated for use in breeding resistance to RLN.

## 5 CONCLUSION

In summary, the absence of well‐established *P. thornei*‐resistant sources in chickpea prompted an extensive evaluation of chickpea accessions across locations. Genotypes including ICC3512, ICC8855, ICC5337, ICC8950, and ICC6537 showed promise for further investigation into specific resistance‐conferring genes. Multi‐trait evaluations, encompassing various biotic and abiotic stresses, highlighted the importance of identifying genotypes with broad‐spectrum resistance. Using advanced GWAS models, FarmCPU outperformed others in controlling *p*‐value inflation. In total, we identified 44 MTAs, with 28 originating from Indian datasets and 16 from Australian datasets. The study's robustness was enhanced by including Australian datasets analyzed with different models. Functional analysis of associated genes identified 24 putative candidates, including those encoding *G6PDH, HSPs*, MYB‐like DNA‐binding protein, zinc finger FYVE protein, and thaumatin‐like proteins, known for their roles in plant‐nematode responses. Notably, the gene *Ca_10016*, associated with hydrolase activity, presented four haplotypes, with haplotypes 1–3 conferring RLN moderate susceptibility and haplotype 4 indicating high susceptibility. These findings open avenues for marker development to enhance breeding for RLN resistance in chickpea, offering a comprehensive understanding of genetic factors and potential targets for crop improvement.

## AUTHOR CONTRIBUTIONS

**Ashish Kumar**: Data curation; investigation; methodology; writing—original draft; writing—review and editing. **Yogesh Dashrath Naik**: Formal analysis; writing—original draft; writing—review and editing. **Vedant Gautam**: Investigation; methodology; writing—review and editing. **Sunanda Sahu**: Investigation; methodology; writing—review and editing. **Vinod Valluri**: Formal analysis; software; writing—review and editing. **Sonal Channale**: Investigation; methodology; writing—review and editing. **Jayant Bhatt**: Resources; writing—review and editing. **Stuti Sharma**: Resources; writing—review and editing. **R. S. Ramakrishnan**: Resources; writing—review and editing. **Radheshyam Sharma**: Resources; writing—review and editing. **Himabindu Kudapa**: Formal analysis; resources; writing—review and editing. **Rebecca S. Zwart**: Investigation; methodology; writing—original draft; writing—review and editing. **Somashekhar M. Punnuri**: Resources; writing—review and editing. **Rajeev K. Varshney**: Formal analysis; resources; supervision; writing—review and editing. **Mahendar Thudi**: Conceptualization; formal analysis; funding acquisition; project administration; supervision; validation; visualization; writing—original draft; writing—review and editing.

## CONFLICT OF INTEREST STATEMENT

The authors declare no conflicts of interest.

## Supporting information


**Supplementary Table 1**: Chickpea mini core collection used in the study.

**Supplementary Table 2**: Descriptive statistics, analysis variance analysis, broad‐sense heritability (H^2^) values for E1 (Jabalpur 2020–21) and E2 (Jabalpur 2021–22) experiment.

**Supplementary Table 3**: Reproduction Factor values in different experiments

**Supplementary Table 4**: Summary and distribution of SNPs on chickpea chromosomes.

**Supplementary Table 5**: Principal component analysis using SNPs identified on 202 chickpea mini core collection.

**Supplementary Table 6**: Summary of significant marker‐trait associations identified using FarmCPU and BLINK models.

**Supplementary Table 7**: Gene annotations of identified MTAs with *Pratylechus thornei* resistance in chickpea.

**Supplementary Table 8**: Haplotypes for the gene *Ca_10016* encoding Metallophosphoesterase present on Ca7

## Data Availability

We have included all relevant data in the manuscript and supplementary files. The codes used for haplotype analysis are available at https://www.bioinformatics.org/sms/iupac.html
